# Let’s Talk About Emotions: the Development of Children’s Emotion Vocabulary from 4 to 11 Years of Age

**DOI:** 10.1007/s42761-021-00040-2

**Published:** 2021-04-16

**Authors:** Gerlind Grosse, Berit Streubel, Catherine Gunzenhauser, Henrik Saalbach

**Affiliations:** 1 Department of Social and Education Sciences¸ Early Childhood Education Studies, Potsdam University of Applied Sciences, Kiepenheuerallee 5, 14469 Potsdam, Germany; 2 Leipzig Research Centre for Early Childhood Development, Leipzig University, Jahnallee 59, Leipzig, 04109 Germany; 3 Department of Educational Sciences, Leipzig University, Marschnerstraße 31, 04229 Leipzig, Germany; 4 Department of Educational Sciences, University of Freiburg, Rempartstraße 11, Freiburg, 79098 Germany

**Keywords:** Lexical acquisition, Children, Cognitive development, Word learning, Emotion-related knowledge, Emotion vocabulary, Conceptual development

## Abstract

Learning to use language in an adult-like way is a long-lasting process. This may particularly apply to complex conceptual domains such as emotions. The present study examined children’s and adults’ patterns of emotion word usage regarding their convergence and underlying semantic dimensions, and the factors influencing the ease of emotion word learning. We assessed the production of emotion words by 4- to 11-year-old children (*N* = 123) and 27 adults (*M* = 37 years) using a vignette test. We found that the older the children, the more emotion words they produced. Moreover, with increasing age, children’s pattern of emotion word usage converged with adult usage. The analysis for semantic dimensions revealed one clear criterion—the differentiation of positive versus negative emotions—for all children and adults. We further found that broad covering emotion words are produced earlier and in a more adult-like way.

## Introduction

Being able to express and share emotions helps children to generate social understanding, empathy, and healthy relationships (Beck et al., 2012; Fabes et al., 2001; Holodynski & Friedlmeier, 2006). Although there is evidence that preverbal infants already have rough expectations about appropriate emotional reactions in specific situations (e.g., Skerry & Spelke, 2014), cultures differ in their conceptualizations of specific emotions (see Russell, 1991) and more specific emotional terms appear only fairly late in development (Widen & Russell, 2008). Theoretical accounts consider language as a representational means serving children to store and access culture-specific emotion-related knowledge, including features and boundaries of emotion categories, causes, consequences, and regulation strategies (Cole et al., 2010; Holodynski et al., 2013; Lindquist et al., 2015). For instance, Lindquist et al. (2015) have suggested that emotion terms help to connect disparate aspects of a culturally relevant emotion category (e.g., bodily sensations, possible situational causes, facial expressions), facilitating its representation as an entity. In line with these theoretical conceptualizations, empirical studies show that a differentiated emotion vocabulary positively relates to various aspects of emotion-related knowledge (Beck et al., 2012; Lindquist et al., 2015; Ornaghi & Grazzani, 2013; Streubel et al., 2020) as well as to emotion concept representation (Nook et al., 2017). Streubel et al. (2020), for example, found that the size of preschool children’s emotion vocabulary (i.e., the range of different emotion words they actively use) predicts their knowledge of emotion regulation strategies (while controlling for general vocabulary). Similarly, Ornaghi and Grazzani (2013) reported that production and comprehension of emotional state language relates to primary school children’s emotion-related knowledge (TEC; Pons & Harris, 2000).

In many domains, learning to connect specific terms to concepts in an adult-like way is a long-lasting process. Children need both a range of different words in a given domain and a comprehension of word meanings entailing knowledge about correct usage, situational aspects, and lexical delimitation (Saji et al., 2011). So far, we know little about how the acquisition of an (adult-like) emotion vocabulary proceeds. The current study examines emotion vocabulary development in 4–11-year-old children using correlation analysis and multidimensional scaling. Moreover, we examined quantitative and qualitative factors—namely input frequency and word specificity—influencing the ease of emotion word learning.

### Domain-Specific Vocabulary Development

In early vocabulary acquisition, children fast map sound-referent relations to rapidly add words to their vocabulary (Markman, 1989; Yu & Smith, 2007). Fast mapping needs only minimal exposure to sound-object pairs (Carey & Bartlett, 1978) and quickly enlarges the active vocabulary. However, fast mapping does not mean that children acquire the adult-like meaning of words (Bloom, 2000; Clark, 2009; Imai et al., 2008). To do this, children have to acquire the relations between words and concepts and the boundaries of concepts within a given domain (Bion et al., 2013). Studies from various domains (e.g., orientation terms; Clark, 1980; verb meanings; Gentner, 1978; Gropen et al., 1991; Saji et al., 2011; container; Ameel et al., 2008; or color terms; Saji et al., 2020) document that it requires many years to attain an adult-like representation of an entire semantic domain. Over these years, children’s patterns of labeling appear to evolve gradually whereby the semantic domain is restructured repeatedly. Saji et al. (2011), for instance, investigated how Chinese-speaking pre- and primary school children used a set of verbs to label various carrying or holding events. Children not only used fewer words but also used them very differently than adults. These results underline that the systematic examination of domain-specific vocabulary development must not only consider how many and which words children have in their vocabulary but also how similar their usage of these words is to that of adults.

### Structure of the Emotion Vocabulary

Emotion vocabulary development poses specific challenges as the domain of emotions is defined less clearly than those of tangible objects (e.g., containers) or perceivable features (e.g., color). Functionalist emotion development accounts propose a hierarchical structure of emotion categories: forming a three-layer hierarchy of superordinate (positive or negative), basic, and subordinate (differentiated and situationally specific; Fischer et al., 1990) categories. In most cultures, the emotion vocabulary seems to organize around few basic concepts—the so-called basic emotions (e.g., *joy*, *fear*, *sadness*; Ekman & Friesen, 1971; Russell, 1991). A similar structure was found for German-language by Schmidt-Atzert & Ströhm (1983). Fischer et al. (1990) propose that developmental pathways of emotion vocabulary follow this hierarchy from basic categories to subordinate ones.

A different approach to investigating emotion concepts and the related terms aims to determine the dimensions by which people perceive the similarities and differences among feelings. A long tradition of emotion studies using a variety of different verbal and non-verbal paradigms has documented a set of two major bipolar dimensions: pleasure-displeasure (also called *valence*) and arousal-sleepiness. Thus, emotion concepts (as indexed by words such as “happiness,” “fear”) are interrelated in a highly systematic fashion. Represented in a two-dimensional space, interrelations form a roughly circular order, the so-called circumplex model of emotion (Russell et al., 1989).

### Emotion Vocabulary Development

Empirical studies show that children use emotional terms from the age of two (Izard & Harris, 1995; Michalson & Lewis, 1985; Ridgeway et al., 1985). Between 3 and 5 years, children start to name basic emotions (Denham, 1998; Harris, 1989). Between 4 and 11 years, emotion vocabulary seems to double every second year, reaching a plateau between 12 and 16 years (Baron-Cohen et al., 2010; Nook et al., 2020). Additionally, in a recent study, Nook et al. (2020) showed that the level of “abstractness” of definitions of emotion words continues to mature up until age 18.

Studies suggest that children acquire emotion words gradually in a certain developmental order. Using free labeling tasks on emotional faces and stories in different samples of 2–5-year-olds, Widen and Russell (2003, 2008) found that *happy*, *angry*, and *sad* emerged early and were used more frequently; *fear*, *surprise*, and *disgust* emerged later and were used less frequently. Children applied early emerging labels more broadly (overgeneralizing), whereas they applied later emerging labels more narrowly.

Li and Yu (2015) found that 2–13-year-old Chinese children comprehend positive emotion words earlier than negative and neutral ones, which might relate to the fact that *valence* is an early distinguishing feature in emotion concept acquisition (Nook et al., 2017). In a study by Russell and Ridgeway (1983) with children and young adults from third grade to undergraduate level, the interrelations among their emotion concepts were explored with multidimensional scaling (MDS) analyses. Results supported the hypothesized circular ordering of emotion words within a two-dimensional space spanning valence and arousal. In their study, the two-dimensional structure accounted for the data of third graders (9 years), almost as well as it did for college students; there were no age differences in the salience of either of the two dimensions. Nook et al. (2017) more recently used MDS analyses to show that *valence* is a key dimension for children’s emotion representations, which develops from a mono-dimensional structure based on *valence* to the common bi-dimensional structure of the circumplex model comprising both *valence* and *arousal* between age 6 and age 25. Thus, while we have consistent results on how the emotion vocabulary develops across childhood, we know little detail about this development.

### Factors Influencing the Ease of Emotion Word Learning

Why do children acquire some emotion words earlier than other ones? We investigate one quantitative and one qualitative factor: (1) *Input frequency* describes how often a certain word is used by caregivers in child-directed speech. Each time children face a specific word being used in context, they have an opportunity to deepen their understanding of the word’s meaning. Empirical studies show that frequently used words are acquired earlier in development (Goodman et al., 2008; Lieven, 2010; Roy et al., 2009). (2) *Word specificity* indicates whether adults use the term consistently to name a specific emotional state or whether they use it broadly for a variety of different states. The more broadly a term is used by adult speakers, the more difficult it might be to determine its meaning and put it to active usage.

### The Present Study

The present study examined 4–11-year-old children’s emotion vocabulary as a connected representational system. Older children should have a larger emotion vocabulary than younger children. However, children can use a specific word without fully capturing its meaning as understood by adults (Ameel et al., 2008; Saji et al., 2011). It is an open question how the pattern of children’s usage of emotion words converges to that of adults and whether children use semantic dimensions to distinguish emotion states in the same way as adults do. Furthermore, we were interested in how input frequency and word specificity influence the ease of emotion word learning.

Using a newly developed vignette test (Streubel et al., 2020), we investigated the following research questions:

1. How many emotion words do children and adults produce across 20 emotional vignettes?
  Hypothesis 1. The older the children, the more emotion words they have in their active vocabulary.
2. Which emotion words do children produce in the different age groups? (*Exploratory Analysis*)
3. How does the pattern of children’s emotion word usage converge to that of adults?
  Hypothesis 3. The older the children, the more similar is their pattern of emotion word usage to that of adults.
4. What are the semantic dimensions underlying the labeling of emotional states depicted in the vignettes by children of the different age groups and adults? (*Exploratory Analysis*)
5. How do input frequency and word specificity of emotion words influence the ease of learning them?
  Hypothesis 5a: The more frequent an emotion word occurs in early language input, the earlier children use the word, but not necessarily in an adult-like way.
  Hypothesis 5b. The more specific a word is the earlier children use the word in their active vocabulary and in a more adult-like way.

## Method

The present study is part of a larger project, which addressed the relations between (general and emotion-specific) language abilities and emotion-related knowledge. Parts of the data have been analyzed for (Streubel et al., 2020). In the present dataset, however, we added an additional age group (10–11-year-old children) and also included children who had been excluded from the previous study due to missing data points in the emotion-related knowledge tasks of the study.

### Participants

A total of 153 German participants took part in this study. We excluded data of one child and two adults from analyses because their native language was not German. The remaining sample consisted of a total of 150 monolingual German participants. Twenty-seven were adults and 123 were children of four age groups: 4- to 5-year-olds (*n* = 30; *M* = 5;1 years; *SD* = 0;4; 10 girls and 20 boys), 6- to 7-year-olds (*n* = 31; *M* = 6;11 years; *SD* = 0;4; 15 girls and 16 boys), 8- to 9-year-olds (*n* = 32, *M* = 8;11 years; *SD* = 0;5; 21 girls and 11 boys), and 10- to 11-year-olds (*n* = 30; *M* = 10;11 years; *SD* = 0;5, 13 girls and 17 boys).

Participants came from a large German city and were recruited via an established database of families who have given their written consent to participate in developmental psychology studies. For reasons of sensitivity about collecting demographic data in Germany, we did not collect data on ethnicity, race, or socioeconomic status from our participants. The official statistics indicate that the population from which participants were drawn consists of 91.0% native Germans and is predominantly middle class (Statistical Office of the Free State of Saxony, 2018).

Additionally, *n* = 27 adults aged 28 to 59 years (*M* = 37;5 years; *SD* = 8;10; 11 females and 16 males) participated in the study. Adult participants were recruited via the website *clickworker.com* and received financial compensation for their participation. The study was performed in accordance with the ethical standards laid down in the Declaration of Helsinki, confirmed by an ethics review board’s approval, and in accord with all applicable laws and rules governing psychological research in Germany.

### Procedure

All children participated in two test sessions, each lasting up to 45 min. They were tested one-on-one by trained female investigators in a quiet room at their preschool, primary school, or the University’s laboratory. The first test session included measures of general and emotion vocabulary. The second session included the emotion-related knowledge tasks (not reported here) and took place at least 1 day and at most 7 days following the first session. Within each session, the tests had a fixed order to minimize potential interference or facilitation effects between tasks.

### Instruments

#### Emotion Vocabulary

**Fig. 1:**
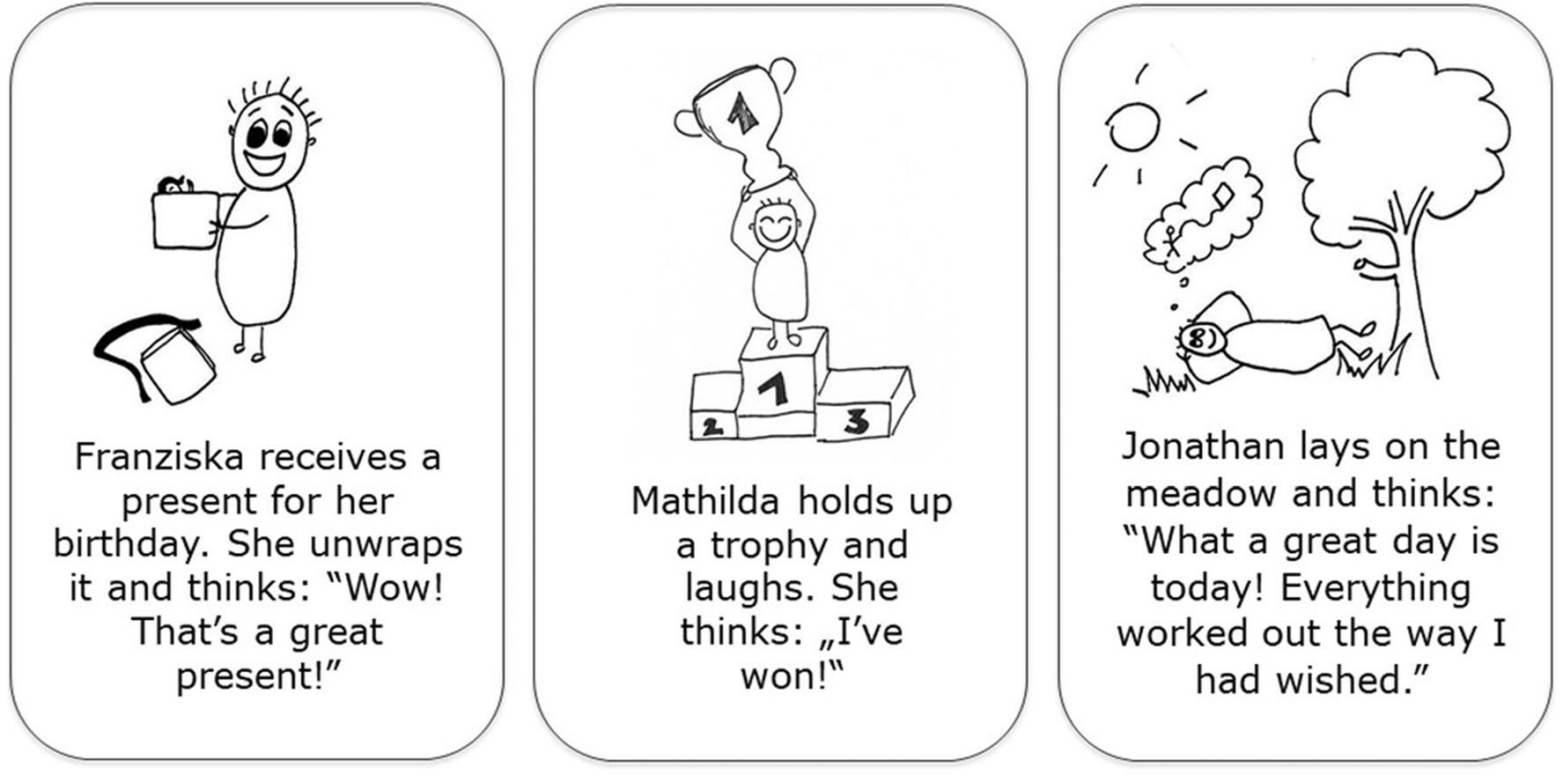
Example vignettes representing joy, pride, and contentment used to assess emotion vocabulary

We used a novel measure, the Children’s Emotion-specific Vocabulary Vignettes Test (CEVVT; for details see Streubel et al., 2020), to assess emotion vocabulary. The CEVVT consists of 20 short illustrated scenarios, each comprising a drawing and an audio-recorded text. Each vignette is tailored to describe a specific emotional state by depicting a child protagonist in a typical emotion-eliciting situation, with emotion-specific facial and bodily expressions, physiological reactions, and thoughts. The 20 vignettes cover the basic emotions joy, fear, sadness, anger, disgust, and surprise as well as 14 complex emotions which are semantically related but subordinated to the basic emotion categories (e.g., pride and contentment as subordinated emotion concepts of joy; frustration and envy as subordinated emotion concepts of anger). The vignettes were developed and selected in a multistage process based on the hierarchical approach of the semantic organization and development of emotion concepts (Fischer et al., 1990; Shaver et al., 1987). In pre-tests, the majority of adults labeled the vignettes with the intended target emotion, a synonymous term, or in case of complex emotions the corresponding basic emotion category. Figure 1 shows examples of the visual stimuli and the according text for the vignettes *joy*, *pride*, and *contentment*.

In a free production task, participants are asked to name the emotion the protagonist in the vignette might feel. This allows for the measurement of number of different emotion words produced and comparison of children’s usage of emotion words with the typical pattern of usage in a representative adult sample. The vignettes were presented on a tablet. After the presentation of each vignette, the children were asked to indicate how the protagonist child in the vignette feels. Depending on the participant’s answer, there were possible follow-up questions. If children did not understand the word *feel*, the investigator asked how the protagonist child is doing. If children answered *well* or *bad*, the investigator asked for clarification (“What do you mean by that? Can you explain in more detail?”). If children described looks or actions (e.g., “laughs,” “cries”), the investigator asked for the accompanying feeling (“How do you feel when you…?”). If children named several emotions, the investigator asked which emotion the protagonist child feels the strongest. A discontinue rule allowed for the termination of the test if children did not know any answer to five consecutive vignettes. However, discontinuation did not lead to exclusion of the subject. This rule was applied to eight children in the youngest age group and one of the 8–9-year-old children. The children’s answers were video-recorded and transcribed. To account for potential fatigue effects, we presented the vignettes in two orders. Each participant started with the same two vignettes (i.e., *joy* and *sadness*). The remaining 18 vignettes were split in half and the order of both halves was counterbalanced among participants. Preliminary analyses revealed no effect of the vignettes’ order on the emotion vocabulary measure. Thus, we did not include vignette order as control variable in the main analyses. It took on average 15 min to complete the test. The development of the vignettes is described in detail in Streubel et al. (2020).

#### Input Frequency (for the 4–5-Year-Old Children Only)

Input frequencies were retrieved from the Childes Database (MacWhinney, 2000), using the German corpora collection via the Stanford “CHILDES-db”-interface (Sanchez et al., 2019). We only used input frequencies for the youngest age group because the entries in the database are mainly for younger children. The database contains entries for children up to 146 months (12 years); but the mean age is *M* = 35.59 months (*SD* = 17.73, min–max = 0.39–146). For children older than 90 months, there are hardly any entries. We used all word forms which contained the respective word stem (see example in footnote^1^) We summed up all entries produced by adult caregivers to children from birth to the age of 5.1 years (i.e. mean age of our youngest age group).

### Data Preparation

#### Coding of Children’s Answers

Two trained persons coded the transcribed answers of the production task. In a first step, the coders indicated whether the child’s answer represents an emotion word or not according to a list we established based on prior research (Baron-Cohen et al., 2010; Schmidt-Atzert & Ströhm, 1983; Shaver et al., 1987; Storm & Storm, 1987). Accordingly, children’s answers were coded as emotion words only, when these or synonymous terms were defined as such in previous studies. Descriptions of physiological states (e.g., tired, hungry), cognitive states or appraisals (e.g., thoughtful, misunderstood, alone), or terms referring to behavior or expressions (e.g., bright) were not coded as emotion words. In a training phase, both coders coded 10% of the dataset (i.e., 5 children per age group). Inter-coder agreement was 98% and disagreement was resolved through discussion.

In a second step, emotion words were categorized based on their common word stem (e.g., *angry* and *anger* were reduced to *angry*). Unspecific positive or negative descriptions of feelings like *good* or *bad*, *not so bad*, or *not good* and negations of specific emotions (e.g., *not sad* or *not happy*) were coded as *unspecific positive* and *unspecific negative* emotion category, respectively.

#### Emotion Word List

For the analyses which are based on the emotion words (e.g., input frequency, word specificity; see analysis 5), we created an emotion word list by extracting those emotion words that were used by at least 5 different participants of the total sample. This list yielded 38 different emotion words, shown in Table 2.

## Analyses and Results

In our analyses of emotion vocabulary, we follow closely the analysis strategy laid out in Saji et al. (2011, 2020). We present the analysis structured by research questions. Descriptions of the measures appear in the respective section.

### Analyses 1: How Many Emotion Words Do Children And Adults Produce Across 20 Emotional Vignettes?

#### Hypothesis 1. The older the children, the more emotion words they have in their active vocabulary.

To examine how many emotion words participants in each age group produced across the 20 vignettes, we calculated the total number of different emotion words used in each age group (see Table 1).

**Table 1:** Number of different emotion words used and similarity of emotion word usage to adult usage in each age group

	Age group				
	4–5 years	6–7 years	8–9 years	10–11 years	Adults
Mean Number of emotion words (SD)	4.4 (1.4)	5.4 (1.1)	7.0 (1.8)	9.7 (2.3)	14.7 (2.4)
Similarity to adult usage^a^	.329***	.405***	.443***	.551***	/

^a^Similarity to adult usage is calculated with Spearman’s ρ; ***p < .001

The adults on average produced 15 different emotion words. These results indicate that adult native speakers of German mostly used different emotion words for each of the 20 emotion vignettes. Children produced a smaller number of different emotion words.

To test for age effects in the mean number of emotion words used, we conducted a one-way-ANOVA with age group as between-subject factor and emotion word number as dependent variable. Age resulted as a significant factor for the variance in the number of emotion words (*F*(4, 145) = 139.9, *p*< .001, *η*^2^ = .79). To scrutinize this result further, we conducted Bonferroni corrected post hoc comparisons for all possible pairings of age groups. The mean number of emotion words differed between all age groups (all *p*s < .01) except between the 4–5-year-old and 6–7-year-old children (*p* = .342). Moreover, the mean number of emotion words of each age group differs significantly from that of adults (all *p*s < .001).

Thus, from the age of 6–7 years onwards, there is a steady, solid increase with age of the number of different emotion words used.

### Analysis 2: Which Emotion Words Do Children Produce in the Different Age Groups?

To examine which emotions words are produced more frequently early on, we analyzed the ranking in frequency of each of the used emotion words in the respective age group.

Table 2 shows the mean production frequency of emotion words. In the youngest age group, the categories *unspecific positive* (18.8% of all labels used) and *unspecific negative* (20.1% of all labels used) were used most frequently. We had a closer look at the non-reduced utterances that children produced in these cases to find out how children name *unspecific positive* and *unspecific negative* emotional states. In the category *unspecific positive*, the top three mentions were *good* [gut], *nice* [schön], and *funny* [lustig]. In the category *unspecific negative*, the top three mentions were *not good* [nicht gut], *bad* [schlecht], and *bad* [böse].

**Table 2:** List of emotion words used by at least 5 participants and their frequencies (in %) and degree of convergence with adult usage per age group as well as input frequency (received from the Childes Database) and word specificity

Emotion word		Production frequency (%)					Degree of convergence with adult usage^a^				Input frequency	Word specificity^b^
English translation	German original	4–5 years	6–7 years	8–9 years	10–11 years	Adults	4–5 years	6–7 years	8–9 years	10–11 years	4–5 years	Adults
Fear	Angst	3.73	5.59	7.50	7.20	8.90	0.47*	0.75**	0.69**	0.85**	323	0.51
Anger	Ärger	0.00	0.00	0.00	1.72	3.49	0.00	0.00	0.00	0.59**	257	0.31
Excited	aufgeregt	0.83	0.00	0.65	0.34	0.78	0.18	0.00	0.18	0.61**	100	0.19
Enthusiastic	begeistert	0.00	0.00	0.00	0.17	0.78	0.00	0.00	0.00	0.51*	21	0.19
Avid	begierig	0.00	0.17	0.00	0.17	0.78	0.00	1.00**	0.00	1.00**	17	0.12
Worried	besorgt	0.21	0.17	0.00	1.72	3.88	0.54*	0.54*	0.00	0.54*	49	0.29
Jealous	eifersüchtig	0.00	0.00	0.16	0.69	0.19	0.00	0.00	1.00**	1.00**	1	0.05
Lonely	einsam	0.62	0.51	0.98	1.72	3.10	0.57**	0.18	0.68**	0.65**	21	0.32
Disgust	Ekel	1.04	1.02	2.12	3.26	3.88	1.00**	1.00**	1.00**	1.00**	65	0.10
Disappointed	enttäuscht	0.00	0.00	0.49	1.37	4.65	0.00	0.00	0.50	0.43	16	0.35
Scared	erschrocken	0.41	2.03	1.63	0.17	0.78	−0.17	0.48*	0.11	0.46*	131	0.21
Joy	Freude	2.28	1.19	1.79	5.32	8.14	0.85**	0.79**	0.98**	0.90**	316	0.74
Glad	froh	3.11	0.51	0.00	0.51	0.00	0.00	0.00	0.00	0.00	70	/
Happy	fröhlich	8.30	17.29	18.92	7.89	0.00	0.00	0.00	0.00	0.00	22	/
Frustrated	frustiert	0.00	0.00	0.00	0.17	0.78	0.00	0.00	0.00	1.00**	2	0.12
Safe	geborgen	0.00	0.00	0.33	1.72	1.94	0.00	0.00	0.69**	1.00**	0	0.16
Annoyed	genervt	0.00	0.00	0.00	0.34	0.97	0.00	0.00	0.00	0.69**	31	0.14
Happy	glücklich	4.767	8.31	5.71	13.21	7.17	0.89**	0.91**	0.94**	0.97**	418	0.89
Horror	Grusel	0.00	0.68	0.00	0.34	0.00	0.00	0.00	0.00	0.00	17	/
Sorrow	Kummer	0.62	0.00	0.33	0.00	0.39	0.33	0.00	−0.11	0.00	6	0.11
Love	Liebe	0.00	0.00	0.00	0.34	0.78	0.00	0.00	0.00	0.65^**^	1349	0.16
Unspecific negative	unspezifisch negativ	20.12	2.71	4.40	2.74	0.58	0.38	0.36	0.33	0.33	619	0.16
Envious	neidisch	0.00	0.00	0.82	1.54	4.26	0.00	0.00	0.73**	1.00**	19	0.07
Embarrassed	peinlich	0.00	0.85	1.63	2.57	2.33	0.00	1.00**	1.00**	1.00**	35	0.16
Unspecific positive	unspezifisch positiv	18.88	8.14	6.36	1.89	0.39	0.40	0.47*	0.53*	0.65**	9092	0.11
Remorse	Reue	0.00	0.00	0.16	0.34	1.94	0.00	0.00	0.73**	1.00**	2	0.24
Angry	sauer	0.62	0.85	1.14	2.23	0.39	0.73**	0.73**	0.50*	0.58*	197	0.08
Shame	Scham	0.00	0.00	0.16	1.20	2.71	0.00	0.00	0.73**	1.00**	13	0.28
Guilty	schuldig	0.00	0.00	0.49	1.37	3.29	0.00	0.00	0.83**	0.84**	14	0.36
Longing	Sehnsucht	0.00	0.00	0.00	0.00	1.36	0.00	0.00	0.00	0.00	6	0.20
Proud	stolz	0.00	0.00	0.49	0.69	1.74	0.00	0.00	0.73**	0.73**	33	0.16
Sad	traurig	31.12	43.22	34.26	28.47	12.40	0.86**	0.91**	0.91**	0.87**	233	1.10
Surprised	überrascht	0.41	0.51	0.65	1.20	3.68	0.42	0.35	0.81**	0.73**	29	0.17
Unhappy	unglücklich	0.62	0.17	0.33	0.17	0.19	0.55*	−0.05	−0.08	−0.05	39	0.05
Desperate	verzweifelt	0.00	0.17	0.00	0.17	0.58	0.00	0.55*	0.00	−0.10	13	0.16
Anticipation	Vorfreude	0.00	0.00	0.00	0.34	0.78	0.00	0.00	0.00	0.73**	0	0.16
Furious	wütend	2.28	5.59	7.99	5.15	2.33	0.84**	0.83**	0.64**	0.65**	11	0.27
Satisfied	zufrieden	0.00	0.00	0.00	0.34	2.71	0.00	0.00	0.00	0.69**	41	0.15

^a^Degree of convergence with adult usage is indicated Spearman’s ρ, *p < .05**; *p* < .01; ^b^word specificity is indicated by the entropy of this word in the adult age group: low values means high specificity, i.e., adults use this word for a small number of vignettes; high values mean low specificity, i.e., adults use this word for a larger number of vignettes; emotion words that were not used by the adult group at all have no entropy value

At age 4–5 years, the most frequently produced basic emotion word was *sad* [traurig] (31.1%), followed by *happy* [fröhlich] (8.3%), and *fear* [Angst] (3.7%).

At age 6–7 years, the most frequently produced emotion words were *sad* [traurig] (43.2%), *happy* [fröhlich] (17.3%), and *happy* [glücklich] (8.3%), followed by *furious* [wütend], and *fear* [Angst] (5.6%, each).

At age 8–9 years, the most frequently produced emotion words were *sad* [traurig] (34.4%), *happy* [fröhlich] (19.0%), *furious* [wütend] (8.0%), *fear* [Angst] (7.5%), and *happy* [glücklich] (5.7%).

At age 10–11 years, the most frequently produced emotion words were *sad* [traurig] (28.5%), *happy* [glücklich] (13.2%), *happy* [fröhlich] (7.9%), and *fear* [Angst] (7.2%).

Thus, *sad* and *happy* (in two forms) are by far the earliest and most frequent emotion words in German children’s emotion vocabulary, according to the present vignette test.

### Analysis 3: How Does the Pattern of Children’s Emotion Word Usage Converge to that of Adults?

#### Hypothesis 3. The older the children, the more similar is their pattern of emotion word usage to that of adults.

To examine how similar children’s pattern of emotion word usage is to adult usage, we created a production matrix for each group separately. In each production matrix, there were 20 rows representing the 20 vignettes and 66 columns representing the emotion words produced across our total sample in response to the 20 vignettes. In each cell of the production matrix, we tallied the number of the participants in the given age group producing the given emotion word for the given vignette.

For each children age group, we calculated Spearman correlations between the given age group’s production matrix and the one of the adult sample; values are 4–5-year-olds: *r*_*s*_ (1320) =.33, *p* < .001; 6–7-year-olds: *r*_*s*_ (1320) =.41, *p* < .001; 8–9-year-olds: *r*_*s*_ (1320) = .44, *p* < .001; 10–11-year-olds: *r*_*s*_ (1320) =.55, *p* < .001 (see also Table 1). To test for differences in the similarity of emotion word usage between age groups, we conducted the test of the difference between two independent correlation coefficients provided by Preacher (2002). Hereby, each correlation coefficient is converted into a *z*-score using Fisher’s *r*-to-*z* transformation. Then, *z*-scores were compared by using the sample size employed to obtain each coefficient (here: *N* = 1,320). This test yielded a significant increase in the similarity of emotion word usage from age 4–5 years to age 6–7 years (*Z* = −2.6, *p* < .05) and from age 8–9 years to age 10–11 years (*Z* = −3.7, *p* < .001). There was no significant increase in the similarity of emotion word usage from age 6–7 years to 8–9 years (*Z* = −1.2, *p* = .117).

Hypothesis 3 can thus be confirmed: The usage pattern of emotion words becomes more similar to adult usage with age. This result indicates that for children, adjusting their labeling of emotional states to adult labeling is a long process, which at 10–11 years is not finished yet.

### Analysis 4: What Are the Semantic Dimensions Underlying the Labeling of Emotional States Depicted in the Vignettes by Children of the Different Age Groups and Adults?

To examine this research question, we adopted the multidimensional scaling (MDS) analyses as used in Malt et al. (1999) and Ameel et al. (2008). MDS provides a geometrical representation of patterns of similarity on dimensions that are extracted to maximize goodness of fit in such a way that inter-point distances on the multidimensional space correspond to dissimilarities between objects. We first created a similarity matrix for each age group. In each matrix, there were 20 rows and 20 columns each representing one of the 20 vignettes. Each cell contained the number of times the given two vignettes were named with the same emotion word.

We conducted MDS for each age group separately. We employed a two-dimensional solution for all age groups as the stress values were near perfect fit for the age groups from 4–5 years to 8–9 years (all stress values < 0.01), excellent for the 10–11-year-olds (stress value < 0.05) and good for the adult age group (stress value < 0.1; Dugard et al., 2010).

**Fig. 2:**
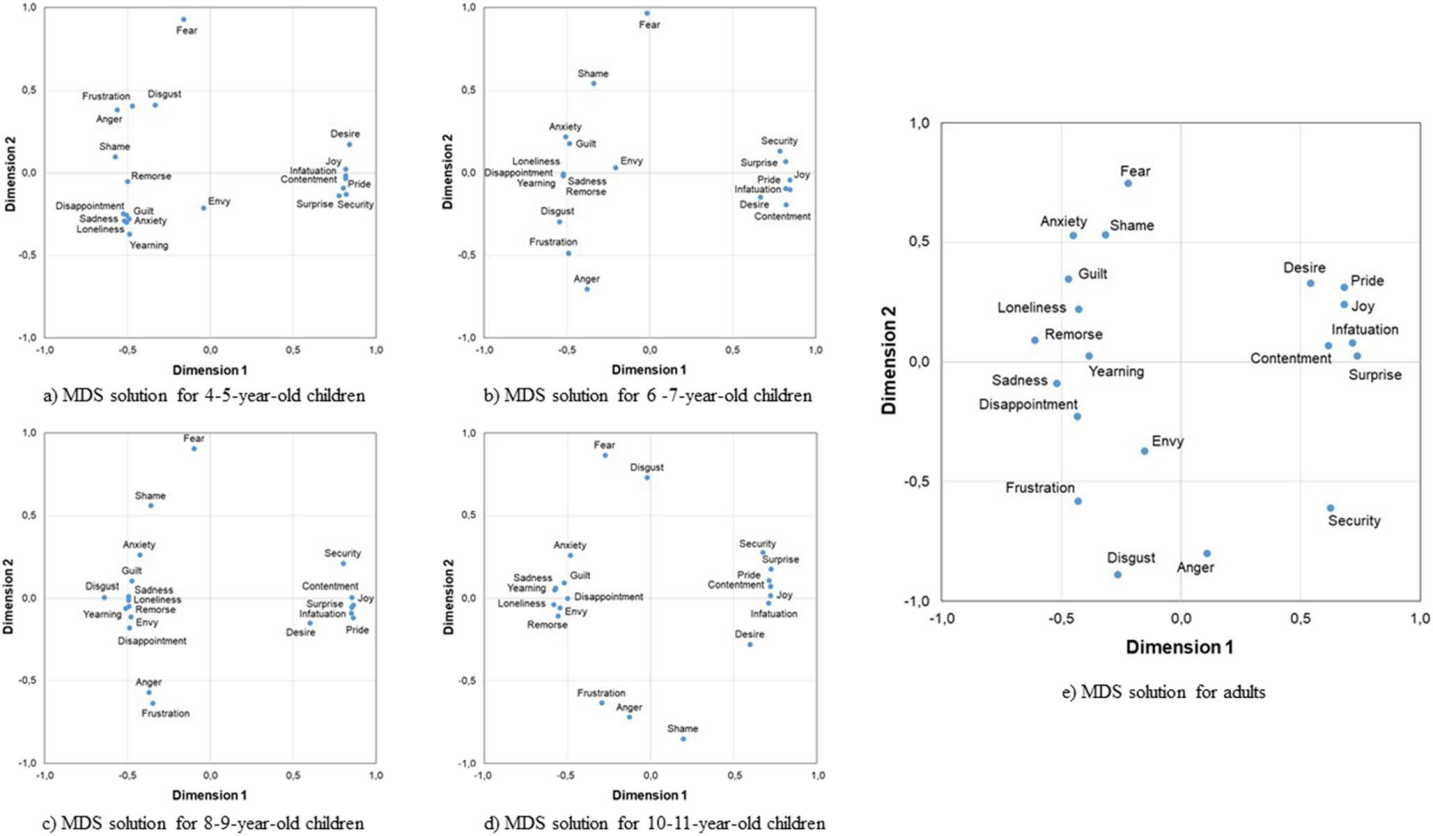
Two-dimensional scaling solutions for the 20 vignettes based on the similarity/dissimilarity of their naming by the children age groups (**a**–**d**) and adults (**e**)

Figure 2 a–e shows the two-dimensional scaling solutions for all age groups. Each point in Fig. 2 a–e represents one of the 20 vignettes, and distances between the points reflect the similarity among the vignettes based on the naming pattern in the respective age group. If participants tended to apply the same emotion word to any given pair of vignettes, the distance between the two vignettes is small, and each of the dimensions extracted reflects a criterion by which the naming of the vignettes is distinguished. The label for the vignette in the figure shows the target emotion word of that vignette.

For the youngest 4–5-year-old children, it can be seen that all the positive emotions cluster together—which means that the corresponding emotional states and their names are not well differentiated. The biggest difference exists between *desire* and *surprise*. Negative emotions are distinguished to a better degree. Especially *fear*, *anger*/*frustration*, *disgust*, *shame*, and *envy* are well separated from the rest including *loneliness*, *sadness*, *yearning*, *disappointment*, and *anxiety*. These other negative emotions are only very little differentiated. On the horizontal axis—dimension 1—positive emotions cluster to the right and negative emotions are dispersed on the left. Dimension 1 represents *valence*. Dimension 2 is less clear and there is quite some movement of single emotions on this axis between age groups.

The configuration of the data points was quite similar for the 6–7-year-old children. Here, *anxiety* and *guilt* start to be separated more clearly. At 8–9 years of age, *sadness*, *yearning*, *remorse*, and *disappointment* are more clearly distinguished from each other. The distances between the points generally increase, indicating a clearer differentiation of emotional states and their names. This tendency continues for the 10–11-year-old children. The horizontal axis still clearly shows the differentiation based on *valence*, with positive emotions on the right and negative emotions on the left hand side.

For the adults, the pattern shows the highest differentiation of all emotional states in space, with negative emotions being more distant from each other than positive ones. Again, the horizontal axis (dimension 1) differentiates positive and negative emotions. Dimension 2 goes from *anger*, disgust, *frustration*, and *security* at the bottom to *anxiety*, *fear*, and *shame* at the top. Whether this second dimension indicates arousal as found in previous research (Feldman Barrett, 2004; Russell et al., 1989) cannot be clearly concluded from the data.

### Analysis 5: How Do Input Frequency and Word Specificity of Emotion Words Influence Their Ease of Learning?

We conducted linear regression analyses separately for each age group, to test the contribution of the two predictors—input frequency and word specificity—to the ease of emotion word learning.

We operationalize *ease of learning* in two different ways: (1) By children’s use of the word in their active vocabulary; operationalized by the *production frequency*, i.e., the mean frequency with which each of the emotion words was produced by the respective age group. (2) By the similarity of usage of the word to adult usage, determined by the *degree of convergence* for each of the emotion words. Here we calculated Spearman correlations between the respective vectors in the *production matrix* of the age group (i.e., the frequency of the emotion word for each vignette; see analysis 3) with the corresponding vectors in the adults’ production matrix (see Table 2 for the distribution of these measure for all emotion words).

*Input frequencies* for the 4–5-year-old children were retrieved from the Childes Database (MacWhinney, 2000), using the German corpora collection via the Stanford “CHILDES-db”-interface (Sanchez et al., 2019). We counted how often children perceived the respective emotion word in the adult speech addressed to them.

*Word specificity* was determined as the *entropy* value of that emotion word in the adult group. The notion of entropy (H) is often used in descriptive statistics as an index to represent the degree of dispersion of responses for a categorical variable. If a response is concentrated in one or a small number of response categories, its entropy value becomes low; if a response is widely dispersed across different response categories, its value becomes high. In the current context, if adults produce a single emotion word for a range of vignettes (i.e., unspecific use), the entropy value of that word will be high. In contrast, if the range of application of the emotion word is restricted to one or a small number of vignettes (i.e., specific use), the entropy value of that emotion word will be low. The respective formula is $H(w)=-\sum \limits_{v=1}^{20}\left\{p(v)\ast logp(v)\right\}$, with *w* = emotion word, and *p*(*V*) = relative frequency with which the word is used for each of the vignettes (*v* = 1 to 20) in the reference data set of the adult participants. Since the entropy measure is not reliable when words are produced by less than five individuals (Mori & Yoshida, 1990), we extracted the emotion words which were produced by at least five individuals.

Table 2 contains the emotion word list and presents the measures, which describe emotion word characteristics.

#### Hypothesis 5a: The more frequent an emotion word occurs in early language input, the earlier children use the word, but not necessarily in an adult-like way.

#### Hypothesis 5b. The more specific a word is the earlier children use the word in their active vocabulary and in a more adult-like way.

Regression models were conducted for each children age group separately for *production frequency* and *degree of convergence* as the dependent measure, respectively. For the youngest age group, multiple regression models were computed with both input frequency and word specificity as predictors. For the other children age groups, single linear regression models were conducted with only word specificity as predictor.

The results of the regression analyses for the *4–5-year-old* children revealed that input frequency and word specificity contributed nearly equally in accounting for *production frequency* and *degree of convergence*. For both *production frequency* and *degree of convergence*, the multiple regression analysis is significant (see Table 3). Regarding *production frequency*, both input frequency and word specificity significantly predict how frequently young children use emotion words in their active vocabulary. For *degree of convergence*, however, word specificity but not input frequency contributes to explaining the variance in the model. Thus, for the 4–5-year-old children, Hypothesis 5a can be confirmed: Words which appear more often in the input are used by children more frequently but not in a more adult-like way.

**Table 3:** Beta values from the regression models for each age group using production frequency and degree of convergence as the dependent measures respectively

	Age group			
	4–5 years	6–7 years	8–9 years	10–11 years
DV: production frequency				
Model fit	*R*^2^ = .40	*R*^2^ = .35	*R*^2^ = .29	*R*^2^ = .63,
	*F*(2, 35) = 13.1 *p* < .001	*F*(1, 36) = 21.3 *p* < .001	*F*(1, 36) = 16.4 *p* < .001	*F*(1, 36) = 64.6 *p* <.001
Input frequency (β)	.471**	/	/	/
Word specificity (β)	.478**	.609***	.559***	.801***
DV: degree of convergence				
Model fit	*R*^2^ = .25, *F*(2, 35) = 7.1, *p* = .003	*R*^2^ = .19, *F*(1, 36) = 9.7, *p* = .004	*R*^2^ = .19, *F*(1, 36) = 9.6 *p* = .004	*R*^2^ = .09, *F*(1, 36) = 4.8, *p* = .032
Input frequency (β)	.134	/	/	/
Word specificity (β)	.528**	.461**	.459**	.344*

*p < .05; **p < .01; ***p < .001

Hypothesis 5b, on the other hand, has to be rejected for the 4–5-year-olds, as our findings rather suggest the reverse pattern: The more unspecific a word is used by the adults (as indicated by a high entropy value), the earlier young children use it in their active vocabulary and the more similar their usage is to that of adults. We discuss this finding below.

Similarly, linear regression analyses in *all other age groups* showed significant effects of word specificity for both *production frequency* and *degree of convergence* (see Table 3 for details). Again, the more unspecific a word is used by the adult age group (as indicated by a high entropy value), the earlier children use it in their active vocabulary and the more similar their usage is as compared to adults. However, the regression coefficient in the models predicting production frequency was much higher than those predicting degree of convergence. We thus conclude that the specificity with which emotion words were used by the adults strongly predicts how frequently children use these words, and—less strongly but still substantially—how easily children up to 11 years of age converge their usage of emotion words to adult usage.

## Discussion

The goal of the present study was to examine children’s emotion vocabulary as a connected representational system across different age groups. This study is, to our knowledge, the first systematical investigation and in-depth analysis of children’s use of emotion words including a wide range of approaches, such as analyzing the frequency of use, the underlying semantic dimensions, and the convergence with adult-like usage as well as the factors influencing the ease of emotion word learning.

We found, that, as expected, children use more different emotion words as they get older. Yet, even for the oldest age group of 10–11-year-old children, the number of emotion words was smaller than that produced by adults. Concerning the question, which emotion words children use early on, we found that children start out with general emotion terms like *good* or *bad* before they use labels of basic emotions like *fear*, *sadness*, or *happiness.* These results are in line with previous studies investigating the development of emotion vocabulary (Baron-Cohen et al., 2010; Denham, 1998; Harris, 1989; Widen & Russell, 2008).

Regarding the question how children’s usage pattern of emotion words converges with that of adults, analyses showed an almost steady increase with age, indicating that it is at 10–11 years far from adult-like. These results document the long developmental course of the structure of emotion vocabulary. This is in line with prior findings by Nook et al. (2017) showing a prolonged development of emotion concepts. The data thus also supports Lindquist et al. (2018) assumption that emotion words and emotion concepts develop in concert.

The analysis of the underlying semantic dimensions revealed two-dimensional solutions. However, only one dimension can clearly be interpreted in all age group samples: the differentiation of positive versus negative emotions. For adults, however, the emotional states are most clearly differentiated from each other distributed in the semantic space. This result confirms previous findings showing a rather mono-dimensional solution for 6–8-year-old children (Nook et al., 2017). However, it diverges from findings which also revealed two-dimensional solutions for all age groups (Russell & Bullock, 1985; Russell & Ridgeway, 1983). Previous findings have mostly reported a structure with *valence* and *arousal* as the two dimensions (Feldman Barrett, 2004; Russell et al., 1989). Our findings can only confirm the valence dimension for children.

The differences between the present findings and prior research might be due to the nature of our stimulus material which was not designed in order to cover all quadrants of the circumplex model (see also Posner et al., 2005), but was instead derived from the hierarchical model of the emotion lexicon by Fischer et al. (1990). Our aim was to represent each of the basic emotion categories identified by that line of research. In two of the previous studies (Feldman Barrett, 2004; Nook et al., 2017), participants provided pairwise ratings of only 10 emotion words, which were partly different from the target emotions which we intended to represent in our study, namely *surprised*, *excited*, *happy*, *calm*, *bored*, *sad*, *upset*, *angry*, *disgusted*, and *scared*. In their designs, participants had to directly compare each emotion pair, while in our study they spontaneously named each of the vignettes either with the same emotion term or with a different one.

We also cannot rule out the possibility that the children age groups and the adult sample differed on other variables due to the cross-sectional design of the study and the fact that the adult sample was recruited in a different way (online) than were children. There might be unknown third variables which drive the differences in the findings between these groups. We suggest, that the present findings of the MDS analysis should be followed up with a different experimental design which ensures same-sized datasets for each age group, e.g., by comparing pairs of emotion words as in previous studies.

A further analysis of the present study provides important implications for a key issue in the literature on vocabulary development: the question of what types of words are easy (or hard) for children to learn. We evaluated how two factors—*input frequency* and *word specificity*—may affect the ease of learning.

*Input frequency* in child-directed speech contributed to explaining emotion word acquisition. Words which were used more frequently in child-directed speech were produced earlier and were used more similarly to adults. This result confirms current theoretical accounts that consider word frequency as an important predictor in accounting for how early the word enters children’s vocabulary (Li et al., 2007). It adds to previous accounts that word specificity also contributed to a high degree in explaining *production frequency.* Particularly, and against our expectation, a lower word specificity leads to a more similar usage. That means, the less specific, i.e., more broad covering, adults use an emotion word, the earlier children start to produce it and the earlier they start to use it also more similarly to adults. This finding is in line with a similar analysis for Chinese carrying/holding events in the study by Saji et al. (2011) who found that children started to use broad covering and frequent verbs the earliest. However, they also reported that use of broad covering verbs tended to converge on adult usage more slowly, whereas our results indicate that broad covering emotion words converge earlier. An explanation for our finding could be (1) that children have more learning opportunities for unspecific emotion words, because they occur more often, and (2) that it is much easier for children to resemble an unspecific usage pattern than it is to narrow in on a very specific usage pattern.

A possible methodological caveat of the present study is that we only have input frequency data for the youngest age group. Input frequencies were retrieved from the CHILDES-db (MacWhinney, 2000), with a limited scope of corpora. More research is needed to confirm our results for the older age groups.

In sum, our results confirm the assumption that the acquisition of the emotion vocabulary poses specific challenges and that children between 4 and 11 years of age are still in the process of differentiating emotional states with words in a similar way as adults. Our results show further that semantic characteristics like *word specificity* might be important factors for word learning that have not yet received much attention in the literature. The present results emphasize the importance of examining children’s word acquisition in terms of quantity, quality, and semantic structure over a long period of development in order to get a fine-grained understanding of vocabulary development. Our study highlights the importance of a systematic investigation of semantic domains as a whole because it gives rise to new and unexpected insights into word learning.

### Additional Information

Note

## Data Availability

Raw data, coding scheme, and analysis material are available under https://osf.io/au8v7/.

## Footnotes

1 Example search string for fear [ängstlich]; ängstlich; änsgtliche, ängstlicher; ängstliches; ängstlichen; ängstlichem; Angst; Ängste; Ängsten; ängstigen; ängstige; ängstigst; ängstigt; ängstigte; ängstigtest; ängstigten; ängstigtet; geängstigt; verängstigen; verängstige; verängstigst; verängstigt; verängstigte; verängstigtest; verängstigten; verängstigtet; verängstigt; verängstigte; verängstigter; verängstigtes; verängstigten; verängstigtem; angstvoll; angstvolle; angstvoller; angstvolles; angstvollen; angstvollem.
